# Effects of differenta mitomycin C concentrations on laser-assisted subepithelial keratectomy

**DOI:** 10.3892/etm.2014.1642

**Published:** 2014-03-28

**Authors:** JIANJIANG SHI, YAOXIN YUAN, SUXIA ZHAO, JIANLONG XU, MENG GUO

**Affiliations:** Department of Ophthalmology, Second Affiliated Hospital of Xingtai Medical College, Xingtai, Hebei 054000, P.R. China

**Keywords:** laser-assisted subepithelial keratectomy, mytomycin C, haze

## Abstract

The aim of the present study was to determine the effect of different concentrations of mitomycin C (MMC) administered intraoperatively during laser-assisted subepithelial keratectomy (LASEK) surgery. A total of 261 patients (496 eyes) were randomly divided into two groups: 0.04% MMC treatment group consisting of 133 patients (245 eyes) and the 0.02% MMC treatment group consisting of 128 patients (251 eyes). The MMC solutions were dropped intraoperatively onto the ablation region and the duration was dependent on the preoperative refractive power of the patient’s cornea: ≤−3.00 diopters (D), 30 sec; between −3.25 D and −6.00 D, 60 sec; between −6.25 D and −9.00 D, 90 sec and ≥−9.25 D, 110 sec. Postoperative observations included haze formation, visual acuity, changes in refractive power, corneal endothelial cell density and incidence of complications. The one year postoperative incidence of haze differed significantly between the groups (P<0.05). The one week and one year comparisons of postoperative visual acuity also differed significantly between the groups (P<0.05). Postoperative changes in corneal refractive power at one, six and 12 months following surgery significantly differed between the groups (P<0.05). No statistically significant difference was identified between the density of corneal endothelial cells prior to surgery and the density at one, six and 12 months following surgery (P>0.05). Thus, the intraoperative application of 0.04% MMC solution effectively inhibited haze formation and markedly improved the efficacy of LASEK surgery, when compared with that of 0.02% MMC.

## Introduction

Laser-assisted subepithelial keratectomy (LASEK) surgery was introduced in 1999 to eliminate the flap-related complications of laser-assisted *in situ* keratomileusis (LASIK) surgery. The LASEK surgical procedure reduces significant postoperative pain, corneal haze and prevents the flap and interface-related problems associated with LASIK surgery (1). Furthermore, LASEK surgery increases visual acuity and decreases aberrations and other defects (2). Patients undergoing LASEK surgery reportedly have reduced risks of postoperative ectasia, indicating that this surface treatment may be safer, particularly when the patients are highly myopic or have thin corneas. However, other complications remain, including haze and regression, which limit the development of LASEK surgery. The present study investigated the postoperative effects of administering different concentrations of mitomycin C (MMC) during LASEK surgery.

## Patients and methods

### Patients

A total of 261 patients were enrolled in the present study. The subjects were aged ≤18 years and were scheduled to undergo LASEK surgery between June 2007 and June 2008. This study was a prospective, randomized, single-center study and all surgical procedures were performed by one surgeon. The 0.04% MMC group comprised 133 patients (245 eyes), including 58 males (112 eyes) and 75 females (113 eyes), aged between 18 and 46 years (mean age, 23.7 years). A total of 28 patients (51 eyes) exhibited low myopia [≤−3.00 diopters (D)], 39 patients (71 eyes) exhibited medium myopia (between 3.25 D and −6.00 D), 31 patients (55 eyes) exhibited high myopia (between 6.25 D and 9.00 D) and 35 patients (68 eyes) exhibited extremely high myopia (≥−9.25 D). The 0.02% MMC group comprised 128 patients (251 eyes), including 61 males (119 eyes) and 67 females (132 eyes), aged between 18 and 44 years (mean age, 22.1 years). A total of 31 patients (59 eyes) exhibited low myopia, 38 patients (76 eyes) exhibited medium myopia, 33 patients (64 eyes) exhibited high myopia and 26 patients (52 eyes) exhibited extremely high myopia. The study was conducted in accordance with the Declaration of Helsinki and approval was obtained from the Ethics Committee of Xingtai Medical College (Xingtai, China). Written informed consent was obtained from all participants.

### Preoperative examination

All patients were subjected to a slit lamp test (SLM-2, Seetool Instrument Co., Ltd., Chongqing, China), a fundoscopic examination (YZ11D Ophthalmoscope and YZ25A Indirect Ophthalmoscope, 66 Vision-Tech Co., Ltd., Suzhou, China), an electronic optometry examination (Accuref-K 9001; Central Medical Ltd., Fukuoka, Japan), a combined optometry examination (Marco CP-690), corneal topography (ATLAS^®^ 995; Carl Zeiss Meditec AG, Oberkochen, Germany), a corneal endothelium cell count, intraocular pressure measurement (AT555, Seetool Instrument Co., Ltd., Chongqing, China) and the corneal thickness was measured via ultrasound (Tomey SP-3000; Tomey Corp., Nagoya, Japan).

### Surgical procedure

All patients were administered topical anesthesia (C_17_H_28_N_2_O_3_·HCl eye drops; twice or three times into the conjunctival sac before surgery; Sam Chun Dang Pharm Co., Ltd., Tokyo, Japan) and received LASEK surgery, according to their optimal preoperative corneal refractive power, via a MEL 80™ Excimer Laser System (Carl Zeiss Meditec AG). The central 8-mm surface region of the cornea was infiltrated with 20% alcohol for 15–25 sec under a microscope and the corneal flap was opened. Following incision using an excimer laser, the surgical area was immediately instilled with 0.02 or 0.04% MMC for a duration that was dependent on the patient’s optimal preoperative corneal refractive power: ≤−3.00 diopters (D), 30 sec; between −3.25 and −6.00 D, 60 sec; between −6.25 and −9.00 D, 90 sec; and ≥−9.25 D, 110 sec. The surgical area was rinsed using physiological saline solution. Following resetting of the corneal flap, a soft corneal contact lens was applied.

### Postoperative medication and follow-up

Tobramycin and dexamethasone eye drops (Guoguang Pharmaceutical Company, Hangzhou, China) were applied four times within 60 min immediately after the surgery and then a further four times in the remaining time of the same day (a total of eight times that day). Then they were administered 7 times the next day, 6 the third day, 5 the fourth day and then 4 times for the next 3 days.

One week following surgery, 0.1% fluorometholone eye drops (Osaka, Japan) were applied four times per day for three weeks, three times per day for three weeks, twice per day for three weeks and once per day for three weeks.

Diclofenac sodium eye drops (0.1%; Hubei Qianjiang Pharmaceutical Co., Ltd., Qianjiang, China) were also administered after the surgery for a week, 4 times per day. Sodium hyaluronate eye drops (Ursapharm Arzneimittel GmbH, Saarbrücken, Germany) were also administered after the surgery, four times per day for whole three months.

Patients were examined once every 2–3 weeks and were followed-up routinely for 1–2 years postoperatively.

### Statistical analysis

The incidence of haze visual acuity was analyzed using a χ^2^ test. Variations in corneal refractive power and corneal endothelial cell counts were analyzed using a t-test. P<0.05 was considered to indicate a statistically significant difference.

## Results

### Treatment

The incidence of haze with 0.04% MMC was 1.22%, while for 0.02% MMC, the incidence was 13.15%. The one year postoperative incidence of haze differed significantly between the groups (P<0.05; Table I). As shown in Table II, the one week and one year comparisons of postoperative visual acuity differed significantly between the groups (P<0.05). The postoperative changes in corneal refractive power at one, six and 12 months following surgery significantly differed between the groups (Table III). No significant difference was identified in the density of corneal endothelial cells prior to surgery or at one, six and 12 months following surgery (P>0.05;Table IV).

### Complications

All patients exhibiting marginal epidermal edema were observed on day 1 and the edema was resolved by day 5. Filamentary keratitis occurred in two patients in the 0.04% MMC group (one bilateral and one unilateral) and in two patients in the 0.02% MMC group (both unilateral).

## Discussion

MMC, an antineoplastic antibiotic produced by *Streptomyces caespitosus*, is a bi- or tri-functional alkylating agent that causes DNA cross-linking and inhibits DNA synthesis. MMC inhibits the growth of numerous types of cell and although MMC is a systemic chemotherapeutic agent, it is commonly applied locally at ophthalmic surgical sites to prevent recurrence, such as in glaucoma, pterygium excision and conjunctival resection (3,4). The activity and biotoxicity of MMC is closely associated with the dosage and usage (5,6). Previous studies identified that the biotoxicity of MMC increased with the dosage and contact time and the effect persisted following treatment (7,8). Biotoxicity of MMC is severe; however, 0.04 mg/ml MMC has been identified as safe and has been used on eye tissues during glaucoma filtration surgery. In the present study, the MMC solution was intraoperatively administered during LASEK surgery. The solution was only in contact with the surface of the cornea and did not affect surrounding cells, thereby minimizing MMC biotoxicity.

Camellin (9) and Virasch *et al* (10) introduced photorefractive keratectomy with an epithelial flap in 1999; this method was subsequently termed LASEK. LASEK was a revision of previous techniques and aimed to improve the postoperative time course and healing process, prevent flap trauma and offer an alternative treatment method for patients with thin corneas. Despite the similarities between LASEK and earlier technologies, the debate surrounding its legitimacy may be irrelevant if results indicate considerable superiority over previous techniques.

LASEK is an important development in refraction correction surgery; however, numerous complications remain, including haze, refractive regression and visual acuity decrease, which restrict further development of this method. Although a number of corneal epithelial cells withstand LASEK surgery and the survival of corneal epithelium cells moderately inhibits haze formation (11,12), haze remains a common complication of various techniques. Previous studies have shown that haze increases the proliferation and activity of corneal cells and results in the irregular arrangement of fibers during novel extracellular matrix synthesis (13). Although LASEK surgery retains the corneal epithelium, ethanol soaks into the matrix system valve plane located between the membrane and the dense plate, triggering postoperative healing of the corneal wound (which normally results in haze formation) (14). Excimer laser surgery is considered to be a stimulus for corneal tissue trauma, therefore, the occurrence of haze is directly associated with wound healing of the corneal tissue (15). The primary method of preventing haze formation is the use of glucocorticosteroids in the ocular region; however, glucocorticosteroid use may result in ocular hypertension, corticosteroid-induced glaucoma and cataracts. Antihyperplasia drugs have been administered in the ocular region to inhibit haze formation (16–19), however, administering MMC in the ocular region inhibits hyperplasia of the corneal epithelium, fibroblasts and collagen fibers, as well as inhibiting haze formation. Furthermore, MMC treatment induces apoptosis of keratocytes and myofibroblasts; however, the mechanism of action underlying the inhibition of haze involves blocking the replication of keratocytes or other myofibroblast progenitor cells (20).

In the present study, two concentrations of MMC were administered during surgery to inhibit haze formation. Corneal haze formation, visual acuity, changes in refractive power, corneal endothelial cell density and corneal healing were observed. The 0.04% MMC group comprised 133 patients, (245 eyes), 58 males (112 eyes) and 75 females (113 eyes) aged between 18 and 46 years (mean age, 23.7 years). A total of 28 patients (51 eyes) exhibited low myopia, 39 patients (71 eyes) exhibited medium myopia, 31 patients (55 eyes) exhibited high myopia and 35 patients (68 eyes) exhibited extremely high myopia. The 0.02% MMC group comprised 128 patients (251 eyes), including 61 males (119 eyes) and 67 females (132 eyes) aged between 18 and 44 years (mean age, 22.1 years). A total of 31 patients (59 eyes) exhibited low myopia, 38 patients (76 eyes) exhibited medium myopia, 331 patients (64 eyes) exhibited high myopia and 26 patients (52 eyes) exhibited extremely high myopia.

In conclusion, the present study indicates that intraoperative application of 0.04% MMC solution effectively inhibits haze formation and markedly improves the outcome of LASEK surgery as compared with the administration of 0.02% MMC.

## Figures and Tables

**Table I:** Incidence of haze one year following surgery (scoring according to the Fantes grading scale).

		Incidence of haze, n			
			
Group	Total eyes, n	Grade I	Grade II	Grade III	Rate, %
0.04% MMC	245	0	3	0	1.22
0.02% MMC	251	18	10	5	13.15

MMC, mitomycin C.

**Table II:** Comparison of postoperative visual acuity with different MMC solutions.

		Optimal visual acuity, n (%)		
		
Group	Total eyes, n	1 week	3 weeks	1 year
0.04% MMC	245	157 (64.08)	245 (100)	243 (99.18)
0.02% MMC	251	180 (72.11)	251 (100)	246 (98.01)

MMC, mitomycin C.

**Table III:** Changes in corneal refractive power measured in diopters (D).

	Eyes exhibiting changes in corneal refractive power		
	
Group	1 month	6 months	1 year
0.04% MMC	−0.52±0.09	−0.29±0.15	−0.35±0.16
0.02% MMC	−0.67±0.11	−0.58±0.21	−0.54±0.18

Values are expressed as mean ± SD. MMC, mitomycin C.

**Table IV:** Density of corneal endothelial cells.

	Density of corneal endothelial cells, cells/mm^2^			
	
Group	Preoperative	Postoperative 1 month	Postoperative 6 months	Postoperative 12 months
0.04% MMC	2,994.01±321.89	2,974.89±339.87	3,001.02±306.92	3,011.18±321.31
0.02% MMC	3,005.92±382.54	2,996.69±374.19	2,988.37±367.33	3,000.05±299.84

Values are expressed as mean ± SD. MMC, mitomycin C.
